# P-1000. The Impact of Re-Initiation of Universal Antenatal Screening on the Incidence of Group B Streptococcus (GBS) Infection

**DOI:** 10.1093/ofid/ofae631.1190

**Published:** 2025-01-29

**Authors:** Nourah Ibrahim Alruqaie, Musaed Alharbi, Hailah Alqarni, Mohammed Alshaalan

**Affiliations:** National Guard Hospital-King Abdullah Specialist Children Hospital, Riyadh, Ar Riyad, Saudi Arabia; National Guard Hospital-King Abdullah Specialist Children Hospital, Riyadh, Ar Riyad, Saudi Arabia; National Guard Hospital-King Abdullah Specialist Children Hospital, Riyadh, Ar Riyad, Saudi Arabia; National Guard Hospital-King Abdullah Specialist Children Hospital, Riyadh, Ar Riyad, Saudi Arabia

## Abstract

**Background:**

Group B streptococcus (GBS) is a leading cause of neonatal bacterial sepsis and meningitis globally. Despite the similar incidence rate of neonatal GBS infection in Saudi Arabia as compared to other countries, there has been a notable increase in Early Onset Disease (EOD) following the discontinuation of universal antenatal screening. Addressing this issue, we evaluated the incidence of EOD after the re-initiation of universal antenatal screening.

Association of gestational age, diagnosis, and mortality with the onset of GBS

**Figure:**
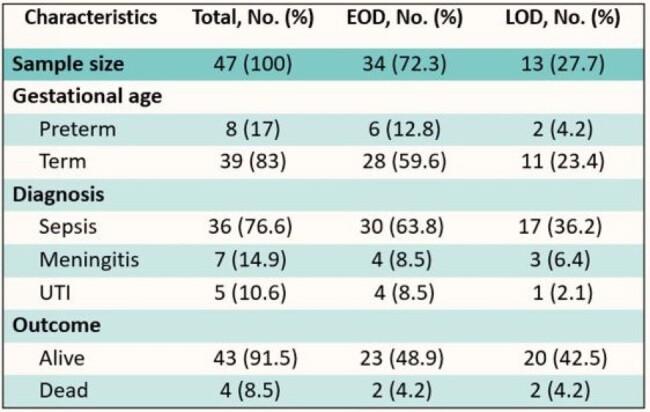

EOD: Early-onset disease

LOD: Late-onset disease

**Methods:**

This study was conducted in a tertiary children's hospital in Riyadh, Saudi Arabia, using a retrospective cohort chart review, which included all cases of neonatal GBS disease identified through microbiology records within the first 90 days of life from January 2017 to December 2023. A standardized form was used to collect maternal and neonatal characteristics by reviewing the charts

**Figure:**
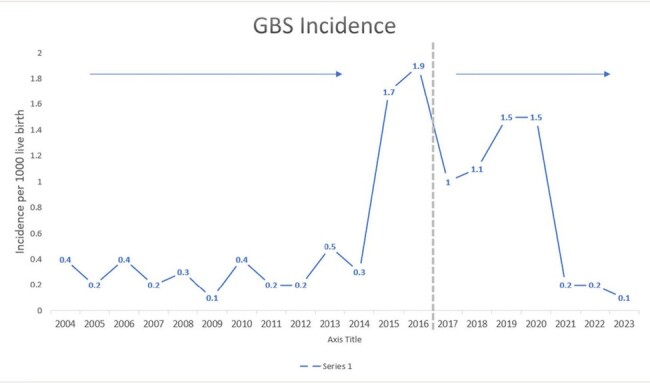

**Results:**

Over seven years, of 61,049 live births, 47 cases of GBS disease were identified (overall incidence, 0.77/1000 live births), and 70% (n = 34) of those had early onset disease (EOD). The annual incidence from 2017 to 2020 showed no significant difference compared to the years when the universal screen was discontinued (2015 and 2016). However, a significant drop was noted in the years after (2021 to 2023), in which the incidence rate was 0.23/1000 live births. We found that 64% (n=30) of mothers who had neonates with GBS disease were either not screened antenatally or were un-booked. Additionally, 49% (n=23) of mothers had a positive GBS screen; however, none of them received appropriate antibiotic prophylaxis. Sepsis was the most common manifestation (76.6%, n = 36), followed by meningitis and Urinary tract infection , 15% and 10.6% respectively. Mortality rate was 8.5% (n = 4)

**Conclusion:**

After three years of re-initiation of universal screening, the incidence of neonatal GBS infection has declined and returned to its previous baseline rate, and similar to worldwide incidence

**Disclosures:**

**All Authors**: No reported disclosures

